# Ectopic expression of the germline transcription factor LSL-1 contributes to developmental delay following failed maternal epigenetic reprogramming

**DOI:** 10.64898/2026.08.28.747910

**Published:** 2026-09-02

**Authors:** Benjamin Nguyen, Zaynab Massenburg, Josh Labus, Sundas Johnson, Sarah D. Blancher, Miyah Jones, Brandon S. Carpenter

## Abstract

Proper transmission of cell identity between generations requires maternal epigenetic reprogramming mechanisms that prevent inappropriate inheritance of lineage-specific transcriptional programs. In *Caenorhabditis elegans* , loss of the H3K4me1/2 demethylase SPR-5 and the H3K9 methyltransferase MET-2 results in ectopic expression of germline genes in somatic tissues and severe developmental delay. Previous studies demonstrated that the chromatin regulator MES-4 contributes to these defects, but whether germline-specific transcription factors also participate in the ectopic transcriptional program remained unclear. Here, we investigated the role of the germline transcription factor LSL-1 in animals lacking SPR-5 and MET-2. We found that genes normally regulated by LSL-1 in the germline are significantly overrepresented among genes ectopically expressed in the soma of *spr-5; met-2* progeny. Consistent with this observation, an endogenously tagged LSL-1 protein became ectopically expressed throughout somatic tissues when maternal SPR-5 and MET-2 activity was disrupted. Furthermore, depletion of LSL-1 partially suppressed the developmental delay observed in *spr-5; met-2* mutants. Transcriptomic analyses revealed extensive overlap between MES-4- and LSL-1-dependent transcriptional programs, with most LSL-1-dependent genes also requiring MES-4. Notably, ectopic expression of *lsl-1* itself depended on MES-4, suggesting that LSL-1 functions downstream of MES-4. Genes dependent on LSL-1 were strongly enriched for germline-associated expression programs and included previously identified direct LSL-1 targets. Together, our findings support a model in which MES-4 promotes ectopic expression of LSL-1, which in turn contributes to a shared germline-associated transcriptional program and developmental delay following failed maternal epigenetic reprogramming. These results demonstrate how lineage-restricted transcription factors cooperate with inherited chromatin states to reinforce aberrant transcriptional programs and disrupt cell fate boundaries.

## Full Text Availability

The license terms selected by the author(s) for this preprint version do not permit archiving in PMC. The full text is available from the preprint server.

